# Bronchiolar chemokine expression is different after single versus repeated cigarette smoke exposure

**DOI:** 10.1186/1465-9921-9-7

**Published:** 2008-01-21

**Authors:** Tomoko Betsuyaku, Ichiro Hamamura, Junko Hata, Hiroshi Takahashi, Hiroaki Mitsuhashi, Tracy L Adair-Kirk, Robert M Senior, Masaharu Nishimura

**Affiliations:** 1 First Department of Medicine, Hokkaido University School of Medicine, Kita-15, Nishi-7, Kita-ku, Sapporo, 060-8683, Japan; 2 Teijin Institute for Bio-medical Research, Teijin Pharma Ltd., 4-3-2 Asahigaoka, Hino, Tokyo 191-8512, Japan; 3 Division of Pulmonary and Critical Care Medicine, Department of Medicine, Washington University School of Medicine and Barnes-Jewish Hospital, 660 So. Euclid Avenue St. Louis, MO 63110, USA

## Abstract

**Background:**

Bronchioles are critical zones in cigarette smoke (CS)-induced lung inflammation. However, there have been few studies on the *in vivo *dynamics of cytokine gene expression in bronchiolar epithelial cells in response to CS.

**Methods:**

We subjected C57BL/6J mice to CS (whole body exposure, 90 min/day) for various periods, and used laser capture microdissection to isolate bronchiolar epithelial cells for analysis of mRNA by quantitative reverse transcription-polymerase chain reaction.

**Results:**

We detected enhanced expression of keratinocyte-derived chemokine (KC), macrophage inflammatory protein-2 (MIP-2), tumor necrosis factor-α (TNF-α), and interleukin-1β (IL-1β) by bronchial epithelial cells after 10 consecutive days of CS exposure. This was mirrored by increases in neutrophils and KC, MIP-2, TNF-α, and IL-1β proteins in the bronchoalveolar lavage (BAL) fluid. The initial inhalation of CS resulted in rapid and robust upregulation of KC and MIP-2 with concomitant DNA oxidation within 1 hr, followed by a return to control values within 3 hrs. In contrast, after CS exposure for 10 days, this initial surge was not observed. As the CS exposure was extended to 4, 12, 18 and 24 weeks, the bronchiolar KC and MIP-2 expression and their levels in BAL fluid were relatively dampened compared to those at 10 days. However, neutrophils in BAL fluid continuously increased up to 24 weeks, suggesting that neutrophil accumulation as a result of long-term CS exposure became independent of KC and MIP-2.

**Conclusion:**

These findings indicate variable patterns of bronchiolar epithelial cytokine expression depending on the duration of CS exposure, and that complex mechanisms govern bronchiolar molecular dynamics *in vivo*.

## Background

Chronic obstructive pulmonary disease (COPD) is characterized by irreversible airflow limitation due to structural alterations of the small airways, chronic inflammation in the airways and alveolar spaces, and loss of elastic recoil caused by destruction of lung parenchyma. Since the pathology of COPD is that of a chronic inflammatory process, many studies have focused on identifying the inflammatory cell types and/or cytokines that play a role in this condition. Increased numbers of neutrophils, macrophages, and lymphocytes in the airways are found associated with COPD [1-3], and various mediators derived from these cells, such as interleukin (IL)-1β, IL-6, IL-8, tumor necrosis factor (TNF)-α, monocyte chemoattractant protein (MCP-1), and matrix metalloproteinase (MMP)-2, MMP-8, and MMP-9, are suggested to contribute to the development of COPD [4,5].

Cigarette smoke (CS) is the main risk factor for the development of COPD. Oxidative stress caused by CS can injure lung cells directly and can trigger cytokine production, leading to the recruitment of inflammatory cells into the lungs [6-8]. The induction of these cytokines is regulated by the activation of redox-sensitive transcription factors, such as nuclear factor-kappa B (NF-κB) [9,10]. Increased expression of NF-κB has been detected in the airway epithelium of smokers compared to non-smokers [11].

Airway epithelium is an important site of cytokine expression in COPD and in response to CS [12,13]. For example, cultured airway epithelial cells produce IL-6 and IL-8 in response to CS exposure [14-16], and TNF-α, IL-8, MCP-1, and macrophage inflammatory protein (MIP)-1α are upregulated in the bronchiolar epithelium of subjects with COPD [17-19]. However, there is scant data on the time course of cytokine responses to CS by airway epithelium. Therefore, we decided to examine the temporal relationship of airway epithelial cytokine production after CS exposure *in vivo *utilizing a mouse model of mainstream CS exposure.

We hypothesized that CS would induce changes in gene expression of pro-inflammatory cytokines, and that the kinetics of the response would differ depending on duration of exposure and the cytokine. Accordingly, we examined the expression of keratinocyte-derived chemokine (KC)/CXCL1 and MIP-2/CXCL2, the combined functional homologues to human IL-8, as well as TNF-α and IL-1β by bronchiolar epithelial cells following either a single CS exposure, repeated exposures for 10 days, or repeated exposure for 24 weeks. We have identified previously unrecognized dynamics in gene expression in bronchiolar epithelium *in vivo *following CS exposure.

## Methods

### CS Exposure

Male C57BL/6J mice, 9–10 weeks of age (Charles River, Atsugi, Japan), were exposed to whole body mainstream CS generated from commercially available filtered cigarettes (12 mg tar/1.0 mg nicotine, Philip Morris, Richmond, VA) by the INH06-CIGR0A smoking system (MIPS Co., Osaka, Japan) using the following parameters: 15.5 puff/min/cigarette; air flow, 0.07 L/min; and volume, 280 mL/second, as described elsewhere [20]. The CS was diluted with filtered air at 1:7 ratio and directed into the exposure chamber (50(L) × 50(W) × 25(H) cm) at a smoke to air ratio of 1:2. The box was fitted with an exhaust vent of the same size as a blower vent in order to avoid the accumulation of mainstream smoke. In initial experiments, mice were exposed to CS for 90 min per day for 1, 3, 7 or 10 days, and were sacrificed 24 hrs after the last CS exposure. For assessment of kinetic patterns in gene expression following CS exposure, mice received either a single 90-min CS exposure or daily exposure for 10 days, and then were sacrificed at 1, 3, 6 or 24 hrs after the last CS exposure. In long-tem CS exposure experiments, mice were exposed to CS for 90 min per day, 6 days per week, for 4, 12, 18 or 24 weeks, and were sacrificed 24 hrs after the last CS exposure. Age-matched, air-exposed mice served as controls. All animal procedures were performed in accordance with the regulations of the Animal Care and Use Committee of Teijin Institute for Bio-medical Research.

### Analysis of plasma cotinine levels

Blood samples were collected at 1 and 3 hrs after the last CS exposure and the levels of cotinine in the plasma were measured using a quantitative enzyme immunoassay kit (Salimetrics, State College, PA), as described previously [21]. Data represent average concentration from 3 mice per condition performed in duplicate.

### Collection of Broncholalveolar Lavage (BAL) fluid

At various times after CS exposure, mice were anesthetized with urethane and α-chloralose and then exsanguinated by severing the abdominal aorta, and BAL fluid was retrieved by injecting 1.0 ml saline through the trachea as described previously [22]. An aliquot of each BAL fluid was mixed with an equal volume of Turk's solution (Wako, Osaka, Japan) and the total cell number was determined using a hemocytometer. Differential cell counts were performed on Diff-Quik™ (International Reagents, Kobe, Japan)-stained cytospin preparations. Data represent the average numbers of cells per ml of BAL fluid from 8 mice per condition. The BAL fluid was centrifuged, and the cell-free supernatants were stored at -80°C until use.

### Detection of albumin, MIP-2, KC, TNF-α, and IL-1β in BAL fluid

The concentration of albumin in BAL fluid was determined using an albumin B test-Wako kit (Wako) according to manufacturer's protocol. The quantity of KC, MIP-2, TNF-α, and IL-1β in the BAL fluid was determined by ELISA kits (R&D Systems, Minneapolis, MN) according to manufacturer's protocols. The detection limit was 7 pg/mL for KC, MIP-2 and IL-1β, and 15 pg/mL for TNF-α. Data represent the average concentration of 8 mice per condition performed in duplicate.

### Immunohistochemical evaluation of DNA oxidation in the lung

Lungs were inflated with diluted Tissue-Tek OCT (Sakura Finetek U.S.A., Torrance, CA) (50% vol/vol in ribonuclease (RNase)-free PBS containing 10% sucrose) and immediately frozen on dry ice as previously described [23]. Antigen retrieval was done on 5 μm sections by incubating in L.A.B. solution (Polysciences, Warrington, PA) at room temperature for 10 min. Sections were incubated with 3% bovine serum albumin (Sigma, St. Louis, MO) and the mouse immunoglobulin blocking reagent from the M.O.M. immunodetection kit (Vector Laboratories, Burlingame, CA) in the TNB solution included in the TSA Biotin System Immunohistochemistry kit (PerkinElmer Life and Analytical Sciences, Wellesley, MA) for 30 min in order to block non-specific binding. Sections were then incubated with the mouse monoclonal anti-8-hydroxy-2'-deoxyguanosine (8-OHdG) antibody (10 μg/mL) (Japan Institute for the Control of Aging, Shizuoka, Japan) for 1 hr at room temperature, followed by 3% hydrogen peroxide for 10 min at room temperature [24]. Immunostaining was developed using the M.O.M. detection kit (Vector Laboratories) with DAB substrate and counterstained with Mayer's hematoxylin.

### Collection of bronchiolar epithelial cells by Laser Capture Microdissection (LCM)

LCM was performed on 7 μm frozen sections to retrieve cells within 100 μm of the bronchoalveolar junction using the PixCell II System (Arcturus Engineering, Mountain View, CA) with the following parameters: laser diameter, 30 μm; pulse duration, 5 ms; and amplitude, 50 mW, as described previously [23]. Approximately 10,000 laser bursts were used to collect cells for RNA isolation from each mouse.

### RNA isolation and real-time RT-PCR

Total RNA was extracted from LCM-retrieved bronchiolar epithelial cells using an RNeasy Mini kit (Qiagen, Hilden, Germany), or from whole lung homogenates using the ISOGEN RNA isolation kit (Nippon Gene Co. Ltd. Toyama, Japan). The quantity and quality of RNA were determined using an RNA LabChip kit (Agilent Technologies, Palo Alto, CA) or a NanoDrop spectrophotometer (NanoDrop Inc., Wilmington, DE). RNA was reverse transcribed using TaqMan Reverse Transcription Reagents kit (Applied Biosystems, Foster City, CA) as described previously [25]. The resulting first-strand cDNAs were used as templates for quantitative real-time RT-PCR using the ABI Prism 7700 Sequence Detector (Applied Biosystems) and gene-specific TaqMan Gene Expression Assays probes (Applied Biosystems) as described previously [18]. Probes for mouse KC (Assay ID: Mm00433859_m1) were derived from the boundary of exons 3 and 4 of the murine KC gene [26]. Probes for mouse MIP-2 (Mm00436450_m1) were derived from the boundary of exons 3 and 4 of the murine MIP-2 gene [27]. Probes for mouse TNF-α (Mm00443258_m1) were derived from the boundary of exons 1 and 2 of the murine TNF-α gene [28]. Probes for mouse IL-1β (Mm00434228_m1) were derived from the boundary of exons 3 and 4 of the murine IL-1β gene [29]. Probes for mouse β2-macroglobulin (β2-MG; Mm00437764_m1) were used as an endogenous control as described previously [25]. The relative amounts of each mRNA in the samples were assessed by interpolation of their cycle thresholds from a standard curve, and were then normalized against β2-MG mRNA. RT-PCR data represent 6–12 mice per condition performed in triplicate.

### Statistical analysis

All results are reported as means ± standard error of the mean (SEM). Statistical significance of the values at each time point after CS exposure was evaluated by Dunnett's type multiple comparative analyses against the values in pretreatment groups. Differences were considered significant at p < 0.05. Statistical analyses were performed using SAS version 8.2 for Windows XP (SAS Institute, Tokyo, Japan).

## Results

### CS Exposure

**Figure 1:**
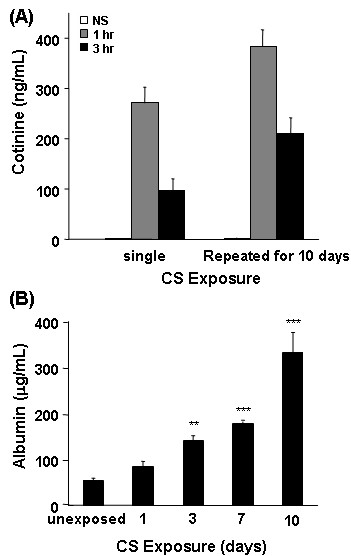
**Plasma cotinine and BAL albumin levels are elevated following CS exposure**. *(A) *Blood samples were collected at 1 and 3 hrs after the last CS exposure and the levels of cotinine in the plasma was measured using a quantitative enzyme immunoassay kit. Data represent average concentration of three mice per condition ± SEM. *(B) *BAL fluids were collected at 24 hr after the last CS exposure and assayed for the presence of albumin using the albumin B test-Wako kit. Data represent the average concentration of eight mice per condition ± SEM. Statistical significance: ** = p < 0.01; *** = p < 0.001.

To confirm adequate CS exposure, the levels of plasma cotinine were measured. Cotinine was essentially undetectable in mice unexposed to CS (<5 ng/mL) (Figure 1A). However, following a single 90-min CS exposure, a dramatic increase in plasma cotinine levels was detected within 1 hr of CS exposure, which was reduced but still elevated 3 hr after CS exposure. Exposure of mice to CS for 10 consecutive days did induce a slight progressive increase of cotinine in the plasma. The levels of plasma cotinine in our studies are similar to that detected in blood samples of ICR mice following CS exposure [30] and in blood samples of humans who smoke >5 cigarettes a day [31].

BAL fluid albumin, a biomarker of tissue injury, was also measured. A significant increase in albumin in the BAL fluid was detected after 3 days of CS exposure, compared to levels in unexposed controls (Figure 1B). The levels of albumin in the BAL fluid continued to increase following 10 consecutive days of CS exposures. These data indicate that the conditions for CS exposure utilized for these studies were sufficient to induce known effects caused by mainstream CS exposure [30,32,33].

### CS-induced DNA oxidative stress in bronchiolar and alveolar epithelium

**Figure 2:**
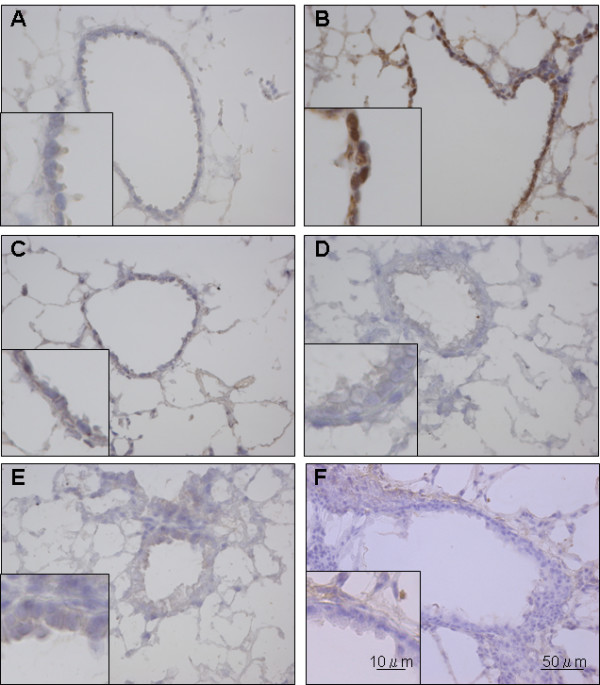
**Initial CS exposure induces oxidative stress in airway epithelial cells**. Mice were unexposed *(A)*, exposed to a single CS exposure *(B and C)*, or repeatedly exposed to CS for 10 days *(D and E)*. Lung sections were stained for oxidative DNA stress using an anti-8-OHdG antibody at 1 hr *(B and E) *or 24 hrs *(C and D) *following the last CS exposure. Normal mouse IgG1 in place of the 8-OHdG antibody served as a negative control *(F)*. Images are representative of five mice per condition.

To determine whether CS exposure induces oxidative stress in lung cells, sections were immunostained for 8-OHdG, a marker of oxidative DNA stress. Oxidative stress was not detected in the lungs of mice unexposed to CS (Figure 2A). Within 1 hr after a single 90-min CS exposure, nuclear staining of 8-OHdG was markedly increased in the bronchiolar and alveolar type II epithelial cells (Figure 2B), confirming that both cell types are major targets of CS oxidants. However, 24 hr after a single CS exposure, the staining was back almost to baseline (Figure 2C). These data are consistent with the findings of Aoshiba *et al*. [34] who examined the kinetics of oxidative stress in mice following a single CS exposure.

Surprisingly, after repeated CS exposure for 10 days, nuclear staining of 8-OHdG was not detected in the bronchiolar or alveolar epithelium either before (Figure 2D) or at 1 hr (Figure 2E) following the final CS exposure. In long-tem CS exposure experiments (4 or 24 weeks), 8-OHdG staining was not observed at 4 or 24 weeks, either (data not shown). Normal mouse IgG1 negative control (DakoCytomation, Glostrup, Denmark) in place of the 8-OHdG antibody resulted in no tissue staining (Figure 2F). These data suggest that repeated CS exposure elicits a mechanism in airway and alveolar epithelial cells to protect against DNA oxidative stress.

### Inflammatory cells in BAL fluid during 10 days of CS exposure

**Figure 3:**
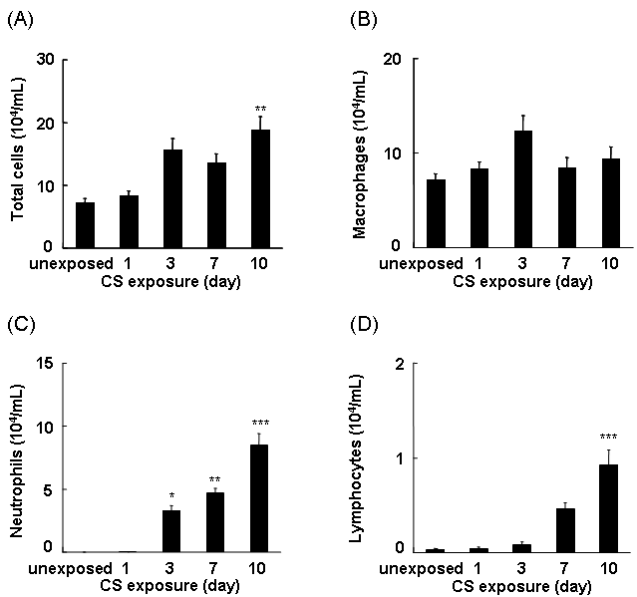
**Repeated CS exposure induces inflammatory cell recruitment**. Mice were repeatedly exposed to CS for up to 10 days and the cell content in the BAL fluid was identified as described in Materials and Methods. Data represent the average number of total cells *(A)*, macrophages *(B)*, neutrophils *(C)*, and lymphocytes *(D) *per ml BAL fluid ± SEM from eight mice. Statistical significance: * = p < 0.05; ** = p < 0.01; *** = p < 0.001.

To determine whether short-term CS exposure elicits an inflammatory response, mice were exposed to CS for up to 10 days and the BAL fluids collected 24 hr after the last CS exposure were examined for the presence of inflammatory cells. After 10 days of CS exposure, the total number of cells in the BAL fluid was significantly increased compared to the BAL fluid of unexposed mice (Figure 3A). Although slightly elevated after 3 days of CS exposure, there was no significant change in the number of macrophages in the BAL fluid irrespective of duration of CS exposure (Figure 3B). In contrast, a significant increase in the number of neutrophils in the BAL fluid was observed after 3 days of CS exposure, which continued to increase following consecutive CS exposures (Figure 3C). A significant increase in the number of lymphocytes was also detected after 10 days of CS exposure (Figure 3D). However, based on the total number of cells relative to the number of each cell type in the BAL fluid, the predominant infiltrating cells in response to CS exposure were neutrophils.

### Neutrophilic chemokines in BAL fluid during 10 days of CS exposure

**Figure 4:**
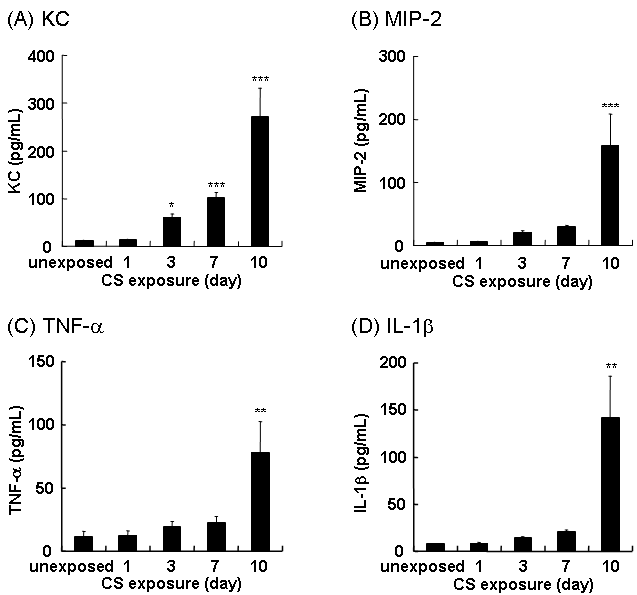
**Repeated CS exposure increases KC, MIP-2, TNF-α and IL-1β in BAL fluid**. Mice were repeatedly exposed to CS for up to 10 days and the levels of KC *(A)*, MIP-2 *(B)*, TNF-α *(C) *and IL-1β *(D) *in the BAL fluid were determined by ELISA. Data represent the average concentration per ml BAL fluid ± SEM from eight mice. Statistical significance: * = p < 0.05; ** = p < 0.01; *** = p < 0.001.

Since the primary infiltrating cells in response to CS exposure were neutrophils, we examined the BAL fluid for cytokines that attract neutrophils. After 3 days of CS exposure, a significant increase in the level of KC in the BAL fluid was observed compared to the BAL fluid from unexposed mice (Figure 4A). The levels of KC in the BAL fluid continued to increase following consecutive CS exposures, paralleling the accumulation of neutrophils in the BAL fluid. A significant increase in the levels of MIP-2 (Figure 4B), TNF-α (Figure 4C) and IL-1β (Figure 4D) was also detected after 10 days of CS exposure.

### Whole lung and bronchiolar cytokine expression during 10 days of CS exposure

**Figure 5:**
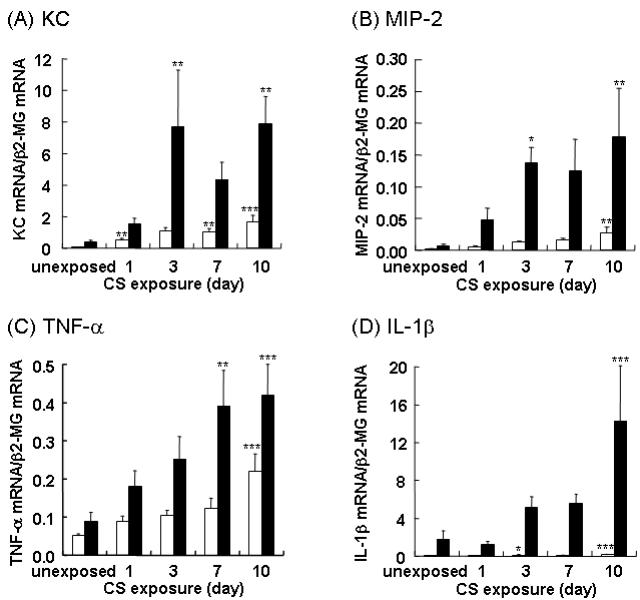
**Repeated CS exposure upregulates KC, MIP-2, TNF-α, and IL-1β expression in whole lung homogenate and in LCM-retrieved bronchiolar epithelium**. Mice were repeatedly exposed to CS for up to 10 days, and the expression of KC *(A)*, MIP-2 *(B)*, TNF-α *(C)*, and IL-1β *(D) *in whole lung homogenates *(white bars) *and LCM-retrieved bronchiolar epithelium *(black bars) *were determined by real-time RT-PCR. Data represent the average expression relative to β2-MG ± SEM from at least six mice. Statistical significance: * = p < 0.05; ** = p < 0.01; *** = p < 0.001.

Since CS produced oxidative stress in the airways (Figure 2), we examined whether bronchiolar epithelial cells express cytokines in response to CS by real-time RT-PCR analyses of RNA isolated from LCM-retrieved terminal bronchiolar epithelial cells. Furthermore, we compared the expression levels of KC, MIP-2, TNF-α, and IL-β in LCM-retrieved bronchiolar epithelial cells to the levels in whole lung homogenates. We found that KC was significantly upregulated after a single CS exposure in whole lung homogenates, whereas a significant upregulation in the bronchiolar epithelium was not detected until following 3 days of CS exposure (Figure 5A). The expression of MIP-2 was increased in bronchiolar epithelial cells after 3 and 10 days of CS exposure (Figure 5B). The expression of TNF-α was increased in bronchiolar epithelial cells after 7 and 10 days of CS exposure (Figure 5C). However, the expression of MIP-2 and TNF-α in whole lung homogenates was not significantly increased until after 10 days of CS exposure. Significant upregulation of IL-1β was observed at 10 days in both whole lung homogenates and in bronchiolar epithelium (Figure 5D). Although there are temporal differences in the expression of these cytokines between whole lung homogenates and bronchiolar epithelium, the expression of these genes was notably higher in bronchiolar epithelial cells when compared with whole lung homogenate at all time points.

### Patterns of bronchiolar cytokine expression after CS exposure

**Figure 6:**
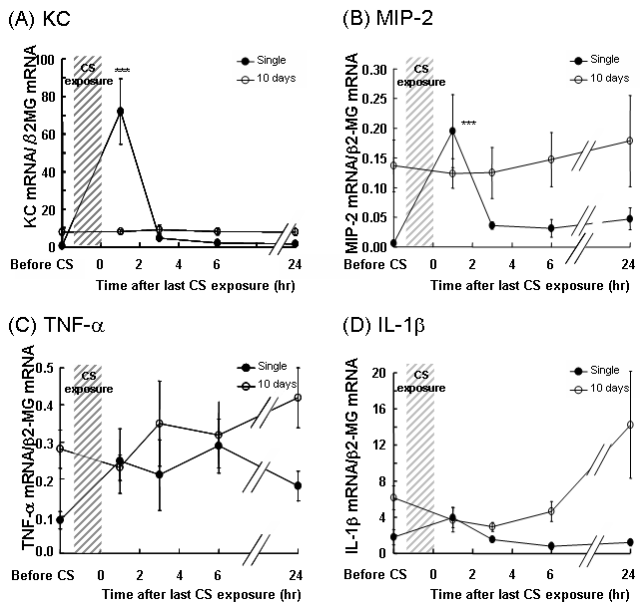
**Kinetics in bronchiolar expression of KC and MIP-2 over 24 hrs is different after single vs. repeated CS exposure**. Mice were exposed to a single CS exposure *(closed circles) *or repeatedly exposed to CS for 10 days *(open circles)*. For the 10 day exposure the time point before CS represents 24 hrs after 9 days exposure. At various times up to 24 hrs following last CS exposure, the bronchiolar epithelial cells were harvested by LCM and the expression of KC *(A)*, MIP-2 *(B)*, IL-1β *(C)*, and TNF-α *(D) *were determined by real-time RT-PCR. Data represent the average expression relative to β2-MG ± SEM from at least six mice. Statistical significance: *** = p < 0.001 vs. before CS exposure at each time point.

To determine the dynamics of the bronchiolar epithelial cell cytokine expression, we examined the expression of KC, MIP-2, TNF-α, and IL-1β by the bronchiolar epithelium over a 24-hr period following either a single CS exposure or repeated exposures for 10 days. In bronchiolar epithelial cells of CS-naïve mice, rapid and robust increases in the expression of KC (70-fold) and MIP-2 (20-fold) were observed within 1 hr of a single CS exposure, compared to unexposed mice (Figure 6A and 6B). These values returned close to baseline values within 3 hrs. Although the expression of KC and MIP-2 in bronchiolar epithelial cells of mice after 10 days of repeated exposure was elevated before the final CS exposure, a transient increase was not observed after CS exposure.

Similar to KC and MIP-2, but to a much lesser extent (2-fold), an increase in IL-1β expression was detected in the bronchiolar epithelium within 1 hr following a single CS exposure which returned close to baseline levels within 3 hrs (Figure 6D). Also similar to KC and MIP-2, the level of IL-1β expression following repeated CS exposure was elevated before the final CS exposure as compared to baseline levels of CS-naïve mice. However, unlike KC and MIP-2, which were not upregulated in response to the final CS exposure, IL-1β expression slowly rose over the 24 hr period following the final CS exposure.

In contrast to KC, MIP-2, and IL-1β, bronchiolar expression of TNF-α failed to return to baseline by 3 hr after the initial CS exposure (Figure 6C) and after 10 days of repeated exposure, there was a slight, slow increase in TNF-α expression by the bronchiolar epithelium following the final CS exposure. These data indicate that the kinetic patterns of expression of different cytokines by the bronchiolar epithelium following CS exposure vary.

### Inflammatory cells in BAL fluid during long-term CS exposure

**Figure 7:**
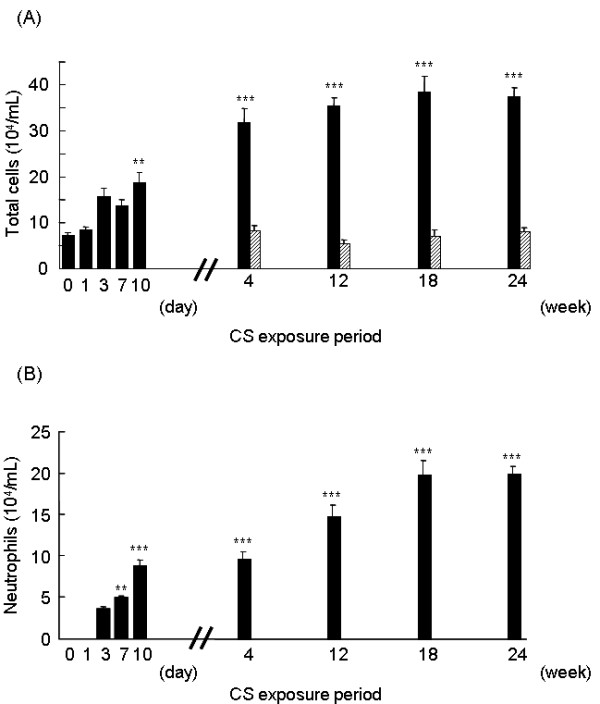
**Long-term of CS exposure induces inflammatory cell recruitment**. Mice were exposed to CS *(black bars) *or to air *(hatched bars) *for 4, 12, 18 and 24 weeks, and the cell content in the BAL fluid was identified as described in Materials and Methods. Data represent the average number of total cells *(A) *and neutrophils *(B) *per ml BAL fluid ± SEM from eight mice. The data set of Fig. 3A and 3C are also included for comparison. Statistical significance: * = p < 0.05; ** = p < 0.01; *** = p < 0.001 vs. before CS exposure at day 0.

Thereafter, we addressed whether the pattern of inflammatory response of the lung to CS exposure observed after 10 days persists following long-term CS exposure. We found that as the exposure of CS to the mice was extended to 4, 12, 18 and 24 weeks, a further increase in the total cell number in BAL fluid was observed (Figure 7A). Similarly, the elevated number of neutrophils in BAL fluid that developed during the short-term CS exposure persisted in the long-term CS exposure, showing over 50% neutrophils out of the total BAL cells at 24 weeks (Figure 7B).

### KC and MIP-2 in BAL fluid during long-term CS exposure

**Figure 8:**
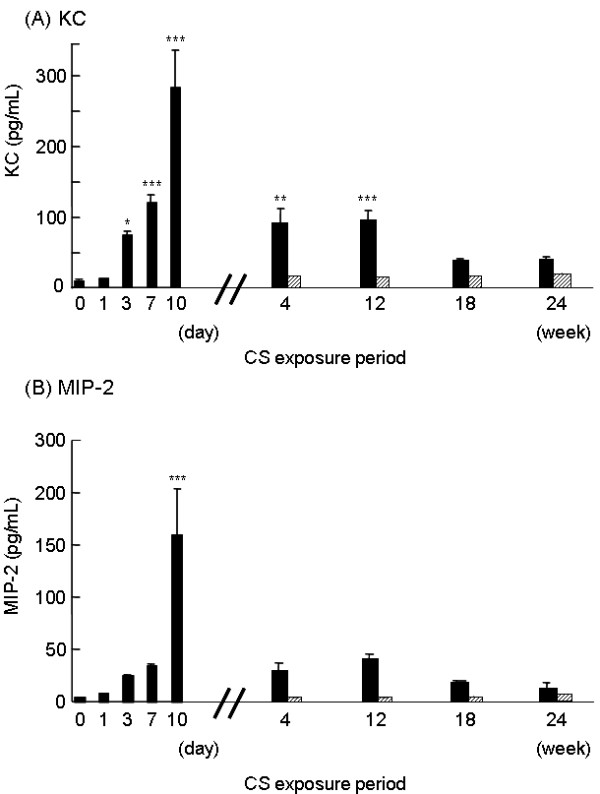
**Long-term of CS exposure does not enhance KC and MIP-2 in BAL fluid**. Mice were exposed to CS *(black bars) *or to air *(hatched bars) *for 4, 12, 18 and 24 weeks and the levels of KC *(A) *and MIP-2 *(B) *in the BAL fluid were determined by ELISA. Data represent the average concentration per ml BAL fluid ± SEM from eight mice. The data set of Fig. 4A and 4B are shown for comparison. Statistical significance: * = p < 0.05; ** = p < 0.01; *** = p < 0.001 vs. before CS exposure at day 0.

In contrast to the parallel increase in the number of neutrophils and the levels of KC and MIP-2 in BAL fluid in the short-term CS exposure experiment, KC and MIP-2 levels in BAL fluid declined by 4 weeks of CS exposure compared to the levels at 10 days despite the persistent increase of neutrophils (Figure 8A and 8B).

### Bronchiolar KC and MIP-2 expression during long-term CS exposure

**Figure 9:**
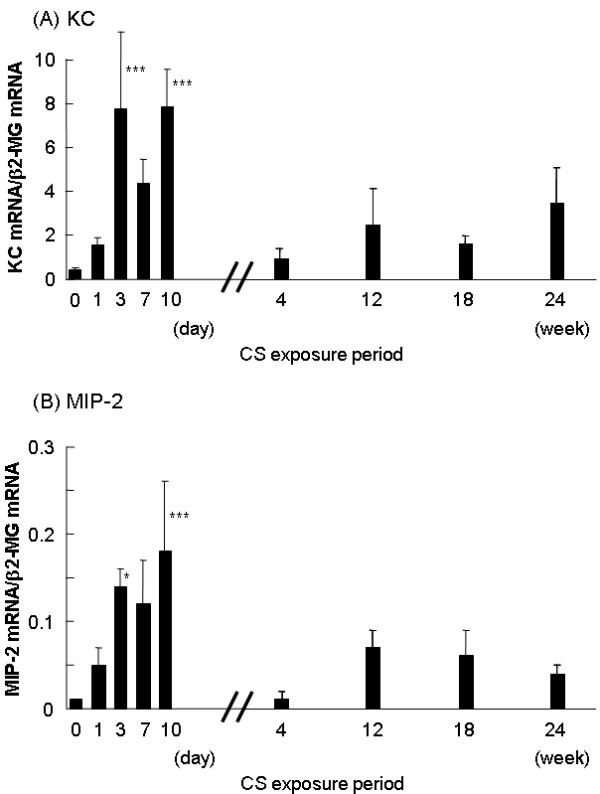
**Long-term CS exposure dampens KC and MIP-2 expressions in LCM-retrieved bronchiolar epithelium**. Mice were exposed to CS for 4, 12, 18 and 24 weeks and the expression of KC *(A) *and MIP-2 *(B) *in LCM-retrieved bronchiolar epithelium *(black bars) *were determined by real-time RT-PCR. Data represent the average expression relative to β2-MG ± SEM from at least six mice. The part of data in Fig. 5A and 5B are also used for comparison. Statistical significance: * = p < 0.05; ** = p < 0.01; *** = p < 0.001 vs. before CS exposure at day 0.

As described above, we detected enhanced bronchiolar expression of KC and MIP-2 after 10 consecutive days of CS exposure (Figure 5A and 5B). However, as the exposure of CS to the mice was extended to 4, 12, 18 and 24 weeks, bronchiolar KC and MIP-2 mRNA were nearly back to baseline after 4 weeks of CS exposure and did not change with continued CS exposure up to 24 weeks (Figure 9A and 9B). Bronchiolar KC and MIP-2 expressions exhibited a similar pattern to those levels in BAL fluid (Figure 8A and 8B).

## Discussion

Prior animal studies have established the expression of pro-inflammatory cytokines in various types of experimental lung injury including CS-induced models [30,35-39]. However, the role of bronchiolar epithelial cells, specifically, in producing pro-inflammatory cytokines and their inflammatory sequela *in vivo *remains to be elucidated. Several approaches might be used to detect cytokine expression. *In situ *hybridization can provide cell-specific information regarding gene expression, but it is not quantitative. Real-time RT-PCR provides quantitative measure of gene expression, but using RNA from homogenized tissue has the disadvantage of averaging-out signals, in that signals from small, but potentially critical, cell populations could go undetected. The use of LCM to selectively isolate a defined cell population improves the sample preparation for gene expression analysis. Furthermore, the predominance of Clara cells in the distal airways of mice [40] enables us to harvest a relatively homogeneous population of cells by LCM and a confirmation method that we harvested distal bronchiolar epithelium, the expression of Clara cell-specific protein (CCSP). In these studies, CCSP expression was more than 6,000-fold higher in LCM-retrieved bronchiolar epithelium compared to that in whole lung homogenate (data not shown). Although the sample was highly enriched in Clara cells, it should be noted that LCM harvests all the cells present at a given site. Thus, migrating inflammatory cells within the bronchiolar epithelium may have influenced the changes in gene expression. However, we detected minimal, if any, Gr-1 stained neutrophils within the bronchiolar epithelial region following 10 days of repeated CS exposure (data not shown). These data suggest that the cytokine expression in the LCM-retrieved samples were derived from the airway epithelium.

The present study indicates that the acute effects of single CS exposure cannot easily be extrapolated to the effects of repeated smoking for short or long term. The effects of CS exposure on bronchiolar epithelial cells over time may result from several processes having different time frames: (a) direct toxic interaction with constituents of CS (including free radicals) that have penetrated the protective antioxidant shield of epithelial lining fluid [41]; (b) damage to cells by toxic reactive products such as hydrogen peroxide generated by interaction between CS and epithelial cells [42] or epithelial lining fluid, which contains oxidized proteins, such as oxidized glutathione and protein carbonyls [43]; and (c) reactions occurring subsequent to the activation of inflammatory-immune processes initiated by (a) and/or (b). Bronchiolar gene expression *in vivo *may thus be affected not only by exogenous CS, but also by the local microenvironment in bronchioles, such as infiltration of inflammatory cells, which cannot be replicated *in vitro*.

CS has been implicated in initiating a lung inflammatory response by activating transcription factors, such as NF-κB and AP-1, and chromatin unwinding (histone acetylation/deacetylation), that lead to upregulation of pro-inflammatory genes [44-46]. Di Stefano *et al*. demonstrated an increase in NF-κB p65 (A) protein in bronchial epithelium from COPD patients and from smokers with normal lung function [11]. Skerrett *et al*. reported that the cell-targeted inhibition of NF-κB activation in distal airway epithelial cells under the Clara cell 10-kDa protein/uteroglobin promoter in mice suppresses the inflammatory response to inhaled lipopolysaccharide, providing direct evidence that NF-κB activation in these cells and the subsequent signal transduction play a critical role in lung inflammation *in vivo *[47]. Elizur *et al*. also demonstrated that Clara cells, a predominant cell type in the distal airways of mice, were the predominant source of KC and MCP-1 in the early response to lipopolysaccharide [48]. In the present study, we observed 8-OHdG formation, a major reactive oxygen species (ROS)-induced DNA stress product, at 1 hr, but not 24 hr after single exposure to CS (Figure 2), which was mirrored by the expression pattern of KC and MIP-2 by bronchiolar epithelial cells (Figure 6). These data suggest that bronchiolar epithelial cells are capable of repairing oxidative DNA stress rapidly, and the temporal DNA stress in bronchiolar epithelial cells is involved in the rapid surge of KC and MIP-2 induction through redox-sensitive transcription factors, such as NF-κB or AP-1. Thus, it should be further investigated how the expression of KC and MIP-2 in those cells following CS challenge is associated with activation of NF-κB and/or AP-1 *in vivo*.

There is apparently marked diversity in the mechanisms of CS-induced inflammatory responses, even between *in vitro *experiments [8,49,50], which precludes further replication of the molecular dynamics in primary cells *in vivo*. It should be noted that rapid bronchiolar induction occurs selectively for KC and MIP-2, but not for TNF-α and IL-1β in response to initial CS exposure, suggesting that these genes are regulated by diverse pathways. All of these cytokines are eventually upregulated in bronchiolar epithelium at 10 days, however, the source of those increased cytokines in BAL fluid would become more complex at later time points, considering many other cell types involved.

In the 10 consecutive day CS exposure experiments, we have found that the response of bronchiolar epithelium to CS varies depending on prior exposure. There are marked differences in the response of the distal airway epithelial cells elicited by the very first CS exposure compared to what happens after repeated CS exposure. After repeated CS exposures, we did not detect DNA oxidative stress or a surge in KC and MIP-2 expression. These data suggest that repeated CS exposure elicits a mechanism in airway epithelial cells to protect against DNA oxidative stress, which in turn affects redox-mediated cytokine production. However, although the surge of KC and MIP-2 expression in response to CS was lost, there was a continual rise in expression of KC, MIP-2, TNF-α, and IL-1β by the bronchiolar epithelial cells upon repeated CS exposure, which was mirrored by their levels in BAL fluids and the influx of neutrophils into the lung up to 10 days.

The comparison of short and long CS exposure models highlighted the complexity of the inflammatory response of the lungs to exposure to CS. The mechanisms by which the long-term CS exposure dampens bronchiolar expressions of KC and MIP-2 need further investigation. Interestingly, neutrophil accumulation in BAL fluid becomes independent of KC and MIP-2 levels during long-term CS exposure. Possible explanations are: (a) other chemoattractants, such as MIP-3α/CCL20, replace KC and MIP-2 to recruit neutrophils [51], (b) extracellular matrix fragments resulting from damage after chronic CS exposure could be pro-inflammatory [52,53], and/or (c) the partitioning of neutrophils between tissue and alveolar spaces changes, possibly due to changes in adhesion and/or development of more channels for neutrophil egress into alveolar spaces.

Taken together, the successful collection of bronchiolar epithelium by LCM and the comparative gene expression analyses has revealed the detailed kinetic profiles of cytokine expression following CS exposure in bronchiolar epithelium. Our data suggest that airway epithelial cells play a role in the recruitment of inflammatory cells in response to CS exposure, and that there are multiple mechanisms by which CS exposure induces cytokine production by bronchiolar epithelial cells. It is to be emphasized that the CS model used in this study is only intended as a bridge between *in vitro *and *in vivo *studies of neutrophil recruitment in response to CS. Extrapolations of current findings to the other experimental CS models or to the human should be made with caution.

## Conclusion

In this study, we described the variable patterns of bronchiolar epithelial cytokine expression depending on the duration of CS exposure, and these findings indicate that complex mechanisms govern bronchiolar molecular dynamics *in vivo*.

## Competing interests

The authors declare that they have no competing interests. The study has not been supported by tobacco industry.

## Authors' contributions

TB conceived of the study, participated in its design, and drafted the manuscript. IH and HT smoked mice to CS, collected lung samples, and carried out ELISA and part of RT-PCR. JH carried out laser capture microdissection and immunohistochemistry and part of RT-PCR, and performed the statistical analysis. TA participated in the study design and drafted the manuscript. HM, RS and MN supervised the study, and helped to draft the manuscript. All authors have read and approved the final manuscript.
